# Cytokine Release Syndrome Following Blinatumomab Therapy

**DOI:** 10.7759/cureus.21583

**Published:** 2022-01-25

**Authors:** Pius E Ojemolon, Sunaina Kalidindi, Taylor A Ahlborn, Osaigbokan P Aihie, Moyosoluwa I Awoyomi

**Affiliations:** 1 Internal Medicine, John H. Stroger, Jr. Hospital of Cook County, Chicago, USA; 2 Internal Medicine, Midwestern University Chicago College of Osteopathic Medicine, Chicago, USA; 3 Medicine, School of Medicine, University of Missouri, Columbia, USA

**Keywords:** acute lymphoblastic leukemia, hematologic malignancy, covid-19, monoclonal antibody, immunotherapy, cytokine storm, cytokine release syndrome

## Abstract

New therapeutic solutions have emerged in the last few decades with the growth and expansion of the field of cancer research. Amongst these new agents, immunotherapy has been prominent, particularly regarding the treatment of hematologic malignancies. One of the most worrisome complications of immunotherapy is cytokine release syndrome (CRS), which represents a supraphysiologic response resulting in excessive release of cytokines and a wide range of systemic manifestations. In this case report, we present a case of cytokine release syndrome following blinatumomab therapy despite premedication with dexamethasone.

## Introduction

American writer Arthur Bloch famously once said, "Every solution breeds many new problems." This perhaps perfectly epitomizes the cytokine release syndrome (CRS), which was first identified and described three decades ago when the anti-T lymphocyte monoclonal antibody muromonab-CD3 (OKT3) was introduced as an immunosuppressive treatment to prevent graft-versus-host disease following solid organ transplantation [1,2].

The cytokine release syndrome is a potentially fatal condition characterized by a systemic response to the exaggerated release of cytokines which occurs in response to immunotherapy. More of a spotlight has been shone on this unique condition given the relative notoriety of its relative, "the cytokine storm," which gained popularity due to the ongoing COVID-19 pandemic. In fact, many authorities regard CRS as a type of cytokine storm. Theoretically, a cytokine storm can be triggered by a myriad of infectious and non-infectious etiologies, hence the term "CRS" is specifically used to describe the cytokine storm that occurs following immunotherapy. Cases of CRS may occur following administration of monoclonal antibodies that target immune effector cells, chimeric antigen receptor (CAR)-T cell therapy, or haploidentical allogeneic hematopoietic cell transplantation [3,4].

Blinatumomab is an anti-CD3/CD19 bispecific T-cell receptor-engaging (BiTE) monoclonal antibody that was approved for the treatment of patients with Philadelphia chromosome-negative B-cell acute lymphoblastic leukemia (ALL) in 2014 and is one of the most well-studied causes of CRS. Recent studies show that 11-15% of patients who receive blinatumomab infusions develop CRS, and 2-5% have severe (grade ≥3) CRS [3,5,6].

## Case presentation

A 41-year-old Hispanic woman with Philadelphia negative B cell ALL was admitted for immunotherapy with blinatumomab, having failed multiple previous chemotherapeutic regimens. She was diagnosed with acute lymphoblastic leukemia at age 39. After screening investigations during a routine antenatal care visit showed marked anemia and leukopenia. Further workup and a bone marrow biopsy confirmed the diagnosis.

She initially received the Cancer and Leukemia Group B (CALGB) 10403 regimen. Following a relapse after completing that regimen, she underwent four cycles of hyperfractionated CVAD (cyclophosphamide, vincristine, Adriamycin, and dexamethasone) and received intrathecal methotrexate and cytarabine. She was then lost to follow-up and returned three months later in a hyperleukocytosis/blast crisis. She was managed in the intensive care unit with leukapheresis and was commenced on the FLAG (fludarabine, Ara-C/cytarabine, G-CSF/filgrastim) regimen. Following the failure of this regimen, a decision was made to use targeted immunotherapy with blinatumomab. The planned regimen was 18 mcg to run over 48 hours on days 1, 3, and 5, 9 mcg to run over 24 hours on day 7, and 28 mcg to run over 24 hours on days 8 to 28. Given the risk of CRS, the regimen included premedication with an intravenous dexamethasone 20 mg bolus prior to blinatumomab infusion on days 1 and 8.

On admission the evening before day 1 of blinatumomab infusion, her blood pressure was 123/76 mmHg, her heart rate was 83 beats per minute, the temperature was 36.6 degrees Celsius, and her oxygen saturation was 97% in room air. Pertinent initial investigations are presented in Table 1.

**Table 1:** Pertinent laboratory results on admission.

Investigation	Result	Reference range
White blood cell count	1,200 cells/µL	4,400–10,600 cells/µL
Absolute neutrophil count	500 cells/µL	2,200–6,900 cells/µL
Blast count	53%	Nil
Platelet count	21,000 cells/µL	161,000–369,000 cells/µL
Hemoglobin	8.8 g/dL	11.7–14.9 g/dL
Hematocrit	25%	34.9–44.3%
Mean corpuscular volume	86.7 fL	81.8–96.9 fL
Mean corpuscular hemoglobin	30.6 pg	25.7–33.2 pg
Mean corpuscular hemoglobin concentration	35.3 g/dL	32.8–35.4 g/dL
Red blood cell distribution width	14.6%	12.3–15.6%

The first six days of the therapy were uneventful, besides a marked decline in blast count to 18% and an increase in the absolute neutrophil count to 1000 cells per µL. However, on day 7, she spiked a fever to 38.7 degrees Celsius and complained of orthostatic dizziness. Her blood pressure was 84/52 mmHg with improvement to 103/61 mmHg after a one-liter bolus of isotonic intravenous fluids. She had a stat chest radiograph and a urinalysis done which showed no concern for pulmonary or urinary tract infection. Two sets of blood cultures (one from a peripheral line and the other from the midline catheter used for the blinatumomab infusion) were also sent, and her blinatumomab infusion was discontinued given concern for grade 2 cytokine release syndrome. She remained hemodynamically stable on maintenance intravenous fluids and her fever resolved three hours after its onset.

Two days later, the blinatumomab infusion was recommenced with the day 7 regimen (9 mcg to run over 24 hours) after intravenous dexamethasone premedication. The next day, she complained of fever and a new-onset rash on her trunk associated with a burning sensation and was hypotensive to 86/47 mmHg. On examination, she had morbilliform, non-blanchable, non-tender, pink patches symmetrically distributed in the upper trunk, sparing the abdomen, groin, and extremities. Repeat workup with concern for possible etiologies for neutropenic fever, similar to those done following her hypotensive episode on day 7, was unrevealing (blood cultures ultimately yielded no growth after five days of incubation). There was a concern for febrile neutrophilic dermatosis, Stevens-Johnson syndrome, and drug reaction with eosinophilia and systemic symptoms (DRESS), but given her background, the blinatumomab infusion was discontinued, marking the termination of the course. She received another one-liter bolus of isotonic intravenous fluids with normalization of her blood pressure afterward, and high-dose intravenous dexamethasone for three days, followed by a four-day taper. Within 48 hours of discontinuation of the blinatumomab infusion, she showed remarkable symptomatic improvement and was discharged home to continue follow-up in the hematology clinic.

## Discussion

CRS is a rare but serious condition that represents a supraphysiologic response to immunotherapy with a resultant overproduction of cytokines from immune effector cells. High tumor burden and profound suppression of hematopoiesis increase the risk of CRS [7,8]. There is no universal definition of high tumor burden in cases of ALL, but it has been described in clinical trials as a bone marrow blast percentage ≥50% [6,9]. At the time of diagnosis, our patient had a bone marrow blast percentage of 90%.

Cytokines involved in CRS include interferon (IFN)-gamma, interleukin (IL)-1, IL-6, IL-8, IL-10, IL-15, monocyte chemoattractant protein (MCP)-1, tumor necrosis factor (TNF) receptor p55, macrophage inflammatory protein (MIP)-1B, and glycoprotein 130 [10]. Recent studies have shed some light on the roles of specific cytokines. IFN-gamma and GM-CSF are products of activated T cells that promote macrophages and monocytes to secrete IL-1 and IL-6, which appear to play very important roles in the downstream processes that lead to the clinical manifestations of CRS [11]. These cytokines can mediate inflammation indirectly by activating prostaglandins. Prostaglandins then promote vasodilation, fever, and hypotension, which are hallmark findings of CRS [7]. IL-6, in particular, is associated with direct mediation of acute inflammation. It induces the expression of VEGF, leading to enhanced angiogenesis and increased vascular permeability. Further endothelial activation can lead to hemodynamic instability and capillary leakage. IL-6 also functions in the bone marrow to increase megakaryocytes and, therefore, platelet production. The combined effect is severe vasodilation and hypotension along with consumptive coagulopathy. These factors lead to the characteristic findings of CRS in susceptible patients [12]. 

CRS presents with a constellation of findings ranging from flu-like symptoms to life-threatening systemic inflammatory responses. Mild cases may present with fever (≥38.0 degree Celcius), fatigue, headache, myalgias, arthralgias, nausea, vomiting, and diarrhea, while more severe cases may demonstrate hypotension, cardiac dysfunction, respiratory distress, pulmonary edema, and multiorgan system failure. Rarely, patients may also exhibit neurotoxicity, manifested as confusion, delirium, aphasia, hallucinations, and altered gait [10,13].

When CRS occurs during a course of blinatumomab, symptoms typically appear during the first infusion cycle but may be delayed by days (as in our case), and the severity of symptoms correlates directly with drug dosage. Overall, the prognosis is good, with 60% of cases reporting fever as the only clinical manifestation and only 2-5% of cases reporting grade 3 toxicity or worse. Diagnosis is based on clinical symptoms in the temporal context of recent blinatumomab infusion. The differential diagnosis includes sepsis, capillary leak syndrome, heart failure, pulmonary embolism, disease progression, tumor lysis syndrome, and macrophage activation syndrome/hemophagocytic lymphohistiocytosis, given the significant overlap in both clinical and laboratory findings with these conditions. In patients with CRS, levels of inflammatory markers, including C-reactive protein (CRP) and ferritin, are often elevated. Studies have shown that CRP values are especially helpful in monitoring patient prognosis. Rising levels of CRP may identify patients at risk for more severe CRS, while declining levels of CRP may be used to identify the peak severity of the syndrome. Levels of cytokines such as IL-6, IL-10, and IFN-gamma may also be elevated, but these tests are unavailable in most centers and the absolute levels of these cytokines may not correlate with the severity of symptoms. In severe cases of CRS, there may be biochemical evidence of tumor lysis syndrome and multiorgan failure [10-14].

Early intervention is critical to prevent progression to life-threatening toxicity. A grading system has been established based on the clinical presentation to guide appropriate management (Table 2). In patients with grade 2 or 3 CRS, temporary infusion interruption is indicated along with symptomatic support. Patients may resume therapy upon symptomatic resolution. However, therapy is re-initiated at low doses and subsequently increased daily. If the blinatumomab infusion is held for more than seven days, a new cycle has to be commenced altogether. Grade 4 toxicity is considered life-threatening and requires permanent discontinuation of therapy with immediate corticosteroid initiation. Therapy may also be discontinued in patients with recurrent ≥ grade 2 CRS or in cases where the infusion is held for more than 14 days. Steroids are the mainstay of treatment for severe BiTe monoclonal antibody therapy-associated CRS. For cases refractory to corticosteroid treatment, tocilizumab, a humanized monoclonal antibody targeted against the IL-6 receptor, has proven to be a useful treatment alternative. Notably, in patients receiving tocilizumab, the reliability of CRP for monitoring inflammation is much reduced as IL-6 blockade itself lowers CRP levels [6,7,13].

**Table 2:** Grading of cytokine release syndrome resulting from BiTe monoclonal antibody therapy. FiO_2_: fraction of inspired oxygen, SBP: systolic blood pressure, ICU: intensive care unit [15].

Grade	Clinical features	Management
1	Fever ± constitutional symptoms	Antipyretics, antihistamines, analgesics, maintenance intravenous fluids.
2	Hypotension responsive to intravenous fluids. Hypoxia corrected by FiO_2 _< 40%.	Intravenous fluid bolus to maintain SBP ≥90 mmHg. Supplemental oxygen to maintain oxygen saturation >90%. Consider treating high-risk patients (advanced age or cardiorespiratory comorbidities) with steroids.
3	Hypotension requiring one pressor. Hypoxia requiring FiO_2_ ≥40%.	Consider ICU admission. Intravenous fluids and supplemental oxygen to meet targets as in grade 2. Low dose pressors. Steroids ± tocilizumab.
4	Life-threatening consequences, e.g., multi-organ failure, respiratory failure requiring ventilatory support, hypotension requiring multiple high dose pressors.	ICU admission. Mechanical ventilation. Multiple high dose pressors. Steroids ± tocilizumab.
5	Death	

Given the risk of life-threatening toxicity from CRS, corticosteroids can be used prophylactically. They can be administered with the first dose of every cycle, before each dose escalation, and when restarting an infusion after treatment interruption. Many treatment regimens with CAR-T cell therapy and BiTe monoclonal antibodies such as blinatumomab now include prophylactic steroids [16,17].

## Conclusions

Increasing application of immunotherapeutic agents is envisaged in the coming years due to their relative successes in cancer treatment. This implies that an increasing incidence of CRS can be expected as well, necessitating a better understanding of the clinical presentation, underlying pathophysiology, and available therapeutic options for this potentially life-threatening condition. Maintaining a high index of suspicion in the appropriate clinical context is of utmost importance to set CRS aside from the most important differential diagnoses. Ongoing research continues to provide better insights into the pathophysiology of CRS and will likely herald the development of more targeted treatment strategies to prevent and treat CRS.
